# Supplementary material for: Structure of the native myosin filament in the relaxed cardiac sarcomere
Source: Nature. 2023 Nov 1;623(7988):863–71. doi: 10.1038/s41586-023-06690-5 (PMC10665186; doi:10.1038/s41586-023-06690-5)
Supplement: Supplementary file 1 — Reporting Summary [file 41586_2023_6690_MOESM1_ESM.pdf]

Corresponding author(s): Stefan Raunser
Last updated by author(s): Aug 30, 2023

Reporting Summary

Nature Portfolio wishes to improve the reproducibility of the work that we publish. This form provides structure for consistency and transparency in reporting. For further information on Nature Portfolio policies, see our Editorial Policies and the Editorial Policy Checklist.

Statistics

For all statistical analyses, confirm that the following items are present in the figure legend, table legend, main text, or Methods section.

n/a	Confirmed	
☐	☒	The exact sample size (*n*) for each experimental group/condition, given as a discrete number and unit of measurement
☒	☐	A statement on whether measurements were taken from distinct samples or whether the same sample was measured repeatedly
☒	☐	The statistical test(s) used AND whether they are one- or two-sided<br>*Only common tests should be described solely by name; describe more complex techniques in the Methods section.*
☒	☐	A description of all covariates tested
☒	☐	A description of any assumptions or corrections, such as tests of normality and adjustment for multiple comparisons
☐	☒	A full description of the statistical parameters including central tendency (e.g. means) or other basic estimates (e.g. regression coefficient) AND variation (e.g. standard deviation) or associated estimates of uncertainty (e.g. confidence intervals)
☒	☐	For null hypothesis testing, the test statistic (e.g. *F*, *t*, *r*) with confidence intervals, effect sizes, degrees of freedom and *P* value noted<br>*Give P values as exact values whenever suitable.*
☒	☐	For Bayesian analysis, information on the choice of priors and Markov chain Monte Carlo settings
☒	☐	For hierarchical and complex designs, identification of the appropriate level for tests and full reporting of outcomes
☒	☐	Estimates of effect sizes (e.g. Cohen's *d*, Pearson's *r*), indicating how they were calculated

Our web collection on statistics for biologists contains articles on many of the points above.

Software and code

Policy information about availability of computer code

Data collection	Cryo-electron tomograms were collected using Serial-EM version 3.8
Data analysis	CTF fitting and motion correction performed in Warp v1.0.9. Tilt series alignment performed in IMOD 3dmod v4.10.51. Tomogram reconstruction and sub-tomogram extraction performed in Warp v1.0.9. Particles picking performed with crYOLO v1.8. Sub-tomogram averaging and post-processing executed in Relion v3.1.0. Map enhancement perfomed with LocSpiral. Tomogram denoising performed with cryoCARE. Protein structure prediction performed with AlphaFold V2.1.1. Segments of the same protein chains were linked together in COOT v0.8.9.2. Flexible fitting molecular dynamic performed with NAMDinator. Figures and videos that depict cryo-EM density maps and protein structures prepared using UCSF ChimeraX v1.5 and ArtiaX extention. Data plotting was done in GrapPad Prism v9 (GraphPad Software).

For manuscripts utilizing custom algorithms or software that are central to the research but not yet described in published literature, software must be made available to editors and reviewers. We strongly encourage code deposition in a community repository (e.g. GitHub). See the Nature Portfolio guidelines for submitting code & software for further information.

Data

Policy information about availability of data

All manuscripts must include a data availability statement. This statement should provide the following information, where applicable:

- Accession codes, unique identifiers, or web links for publicly available datasets
- A description of any restrictions on data availability
- For clinical datasets or third party data, please ensure that the statement adheres to our policy

Cryo-ET structures have been deposited to the Electron Microscopy Data Bank (EMDB) under accession numbers (dataset in brackets): EMD-18200 (thin filament consensus map) [https://www.ebi.ac.uk/emdb/EMD-18200], EMD-16986 (thin filament with masked out tropomyosin) [https://www.ebi.ac.uk/emdb/EMD-16986], EMD-16987 (thin filament including tropomyosin) [https://www.ebi.ac.uk/emdb/EMD-16987], EMD-18147 (thin filament composite map) [https://www.ebi.ac.uk/emdb/EMD-18147], EMD-16991 (M-band from the relaxed thick filament) [https://www.ebi.ac.uk/emdb/EMD-16991], EMD-16993 (Crown P1 from the relaxed thick filament) [https://www.ebi.ac.uk/emdb/EMD-16993], EMD-16990 (Crowns P2-A1 from the relaxed thick filament) [https://www.ebi.ac.uk/emdb/EMD-16990], EMD-16997 (Crowns A1-A5 from the relaxed thick filament) [https://www.ebi.ac.uk/emdb/EMD-16997], EMD-16996 (Crowns A5-A7 from the relaxed thick filament) [https://www.ebi.ac.uk/emdb/EMD-16996], EMD-16995 (Crowns A8-A12 from the relaxed thick filament) [https://www.ebi.ac.uk/emdb/EMD-16995], EMD-16994 (Crowns A11-A15 from the relaxed thick filament) [https://www.ebi.ac.uk/emdb/EMD-16994], EMD-16992 (Crowns A15-A29 from the relaxed thick filament) [https://www.ebi.ac.uk/emdb/EMD-16992], EMD-18146 (Helical reconstruction of the C-zone from the relaxed thick filament) [https://www.ebi.ac.uk/emdb/EMD-18146], EMD-18198 (Helical extrapolation of the C-zone from the relaxed thick filament) [https://www.ebi.ac.uk/emdb/EMD-18198].
Representative tomograms have been deposited under accession numbers EMD-16989 (Tomogram of sarcomere M-band to C-zone from mouse cardiac muscle) [https://www.ebi.ac.uk/emdb/EMD-16989] and EMD-16988 (Tomogram of sarcomere C-zone from mouse cardiac muscle) [https://www.ebi.ac.uk/emdb/EMD-16988].

The atomic coordinates of the protein structures have been submitted to the Protein Data Bank under accession codes (dataset in brackets): 8Q4G (Thin filament from the relaxed mouse cardiac muscle) [https://doi.org/10.2210/pdb8Q4G/pdb], 8Q6T (Thick filament helically reconstructed from the C-zone of the relaxed mouse cardiac muscle) [https://doi.org/10.2210/pdb8Q6T/pdb]. We used the following previously published structures for modelling and comparisons: 5TBY and 6KN7. Source data are provided with this paper.

Human research participants

Policy information about studies involving human research participants and Sex and Gender in Research.

Reporting on sex and gender	*Use the terms sex (biological attribute) and gender (shaped by social and cultural circumstances) carefully in order to avoid confusing both terms. Indicate if findings apply to only one sex or gender; describe whether sex and gender were considered in study design whether sex and/or gender was determined based on self-reporting or assigned and methods used. Provide in the source data disaggregated sex and gender data where this information has been collected, and consent has been obtained for sharing of individual-level data; provide overall numbers in this Reporting Summary. Please state if this information has not been collected. Report sex- and gender-based analyses where performed, justify reasons for lack of sex- and gender-based analysis.*
Population characteristics	*Describe the covariate-relevant population characteristics of the human research participants (e.g. age, genotypic information, past and current diagnosis and treatment categories). If you filled out the behavioural & social sciences study design questions and have nothing to add here, write "See above."*
Recruitment	*Describe how participants were recruited. Outline any potential self-selection bias or other biases that may be present and how these are likely to impact results.*
Ethics oversight	*Identify the organization(s) that approved the study protocol.*

Note that full information on the approval of the study protocol must also be provided in the manuscript.

Field-specific reporting

Please select the one below that is the best fit for your research. If you are not sure, read the appropriate sections before making your selection.

☒ Life sciences ☐ Behavioural & social sciences ☐ Ecological, evolutionary & environmental sciences

For a reference copy of the document with all sections, see nature.com/documents/nr-reporting-summary-flat.pdf

Life sciences study design

All studies must disclose on these points even when the disclosure is negative.

Sample size	We collected 89 tomographic volumes. Sample sizes for the ten cryo-EM map obtained in this study: structure of F-actin without tropomyosin resulted from the averaging of 100,447 particles, structure of F-actin with tropomyosin resulted from the averaging of 100,447 particles,

	structure of the thick filament M-band resulted from the averaging of 846 particles, structure of the thick filament Crown P1 resulted from the averaging of 1001 particles, structure of the thick filament Crowns P2-A1 resulted from the averaging of 1001 particles, structure of the thick filament Crowns A1-A5 resulted from the averaging of 1261 particles, structure of the thick filament Crowns A5-A7 resulted from the averaging of 1261 particles, structure of the thick filament Crowns A8-A12 resulted from the averaging of 1261 particles, structure of the thick filament Crowns A11-A15 resulted from the averaging of 1092 particles, structure of the thick filament Crowns A15-A29 resulted from the averaging of 5915 particles.
Data exclusions	During the cryo-EM image processing, particles that represented false picks were discarded through 2D and 3D classification procedures. This process, which is required to obtain high-resolution reconstructions, is a standard procedure in cryo-EM image processing. Duplicated particles were removed.
Replication	Cryo-EM grids of vitrified material were obtained in a single plunging session. It is unattainable from a time and cost perspective to repeat cryo-EM data collection and processing on the exact same sample.
Randomization	For the 3D refinement of cryo-EM structures, particles were randomly split into two half sets. For all other experiments, randomization was not required because all data were used in the analysis. Covariates were not controlled.
Blinding	This study does not involve any experiments where blinding would be applicable.

Reporting for specific materials, systems and methods

We require information from authors about some types of materials, experimental systems and methods used in many studies. Here, indicate whether each material, system or method listed is relevant to your study. If you are not sure if a list item applies to your research, read the appropriate section before selecting a response.

Materials & experimental systems

n/a	Involved in the study
☐	☒ Antibodies
☒	☐ Eukaryotic cell lines
☒	☐ Palaeontology and archaeology
☐	☒ Animals and other organisms
☒	☐ Clinical data
☒	☐ Dual use research of concern

Methods

n/a	Involved in the study
☒	☐ ChIP-seq
☒	☐ Flow cytometry
☒	☐ MRI-based neuroimaging

Antibodies

Antibodies used	Affinity-purified rabbit polyclonal antibody against titin kinase
Validation	Antibody was affinity-purified against recombinant rat titin kinase and validated by Western blot against recombinant titin fragments containing the kinase domain as well as negative controls (fragments not containing the kinase outside of the P-zone/M-band transition).

Animals and other research organisms

Policy information about studies involving animals; ARRIVE guidelines recommended for reporting animal research, and Sex and Gender in Research

Laboratory animals	BALB/c mice were used for myofibril preparations.
Wild animals	*Provide details on animals observed in or captured in the field; report species and age where possible. Describe how animals were caught and transported and what happened to captive animals after the study (if killed, explain why and describe method; if released, say where and when) OR state that the study did not involve wild animals.*
Reporting on sex	Sex was not considered as there is no evidence that myofibril structure differs between male and female.
Field-collected samples	*For laboratory work with field-collected samples, describe all relevant parameters such as housing, maintenance, temperature, photoperiod and end-of-experiment protocol OR state that the study did not involve samples collected from the field.*
Ethics oversight	Animals were sacrificed in a schedule-1 procedure by cervical dislocation following licensed procedures approved by King's College London ethics committee and the Home Office UK.

Note that full information on the approval of the study protocol must also be provided in the manuscript.
